# Three Huntington’s Disease Specific Mutation-Carrying Human Embryonic Stem Cell Lines Have Stable Number of CAG Repeats upon *In Vitro* Differentiation into Cardiomyocytes

**DOI:** 10.1371/journal.pone.0126860

**Published:** 2015-05-20

**Authors:** Laureen Jacquet, Andreas Neueder, Gabor Földes, Panagiotis Karagiannis, Carl Hobbs, Nelly Jolinon, Maxime Mioulane, Takao Sakai, Sian E. Harding, Dusko Ilic

**Affiliations:** 1 Stem Cell Laboratory, Assisted Conception Unit, Division of Women’s Health, King’s College London, Guy's Hospital, London, SE1 9RT, United Kingdom; 2 Division of Genetics and Molecular Medicine, King's College London, Guy's Hospital, London, SE1 9RT, United Kingdom; 3 National Heart and Lung Institute, Imperial College, ICTEM, 4th Floor, Hammersmith Campus, Du Cane Rd, London, W12 0NN, United Kingdom; 4 Histology Laboratory, Wolfson Centre for Age-Related Diseases, King's College London, London, SE1 1UL, United Kingdom; 5 Department of Molecular and Clinical Pharmacology, Institute of Translational Medicine, The University of Liverpool, Sherrington Building, Ashton Street, Liverpool, L69 3GE, United Kingdom; University of Newcastle upon Tyne, UNITED KINGDOM

## Abstract

Huntington disease (HD; OMIM 143100), a progressive neurodegenerative disorder, is caused by an expanded trinucleotide CAG (polyQ) motif in the *HTT* gene. Cardiovascular symptoms, often present in early stage HD patients, are, in general, ascribed to dysautonomia. However, cardio-specific expression of polyQ peptides caused pathological response in murine models, suggesting the presence of a nervous system-independent heart phenotype in HD patients. A positive correlation between the CAG repeat size and severity of symptoms observed in HD patients has also been observed in *in vitro* HD cellular models. Here, we test the suitability of human embryonic stem cell (hESC) lines carrying HD-specific mutation as *in vitro* models for understanding molecular mechanisms of cardiac pathology seen in HD patients. We have differentiated three HD-hESC lines into cardiomyocytes and investigated CAG stability up to 60 days after starting differentiation. To assess CAG stability in other tissues, the lines were also subjected to *in vivo* differentiation into teratomas for 10 weeks. Neither directed differentiation into cardiomyocytes *in vitro* nor *in vivo* differentiation into teratomas, rich in immature neuronal tissue, led to an increase in the number of CAG repeats. Although the CAG stability might be cell line-dependent, induced pluripotent stem cells generated from patients with larger numbers of CAG repeats could have an advantage as a research tool for understanding cardiac symptoms of HD patients.

## Introduction

Huntington’s disease (HD; OMIM 143100) is an autosomal, dominantly inherited progressive neurodegenerative disorder usually with a late onset. It is caused by an expanded polymorphic polyglutamine (polyQ) trinucleotide (CAG) motif in the first exon of the *HTT* gene. *HTT* encodes huntingtin (HTT), a large 348 kD protein ubiquitously expressed, with highest levels found in the brain and testis [1–4]. HTT endogenous function is still not completely understood as it has very little homology to other known proteins [1].

In healthy individuals, the CAG repeat number ranges from 11 to 34 while numbers greater than 36 are causative of HD. The number of repeats generally determines age of disease onset [1, 5, 6]. Individuals with over 55 CAG repeats tend to develop Juvenile Huntington’s Disease (JHD), a more severe form, with slightly different clinical manifestations that develop in their youth instead of in their third to fifth decade. HD patients bearing homozygous mutations do not automatically have a lower age of onset, but do have a more severe phenotype and disease progression.^6^ The mutation shows anticipation, with both decreases and increases in repeat length occurring upon parent to offspring transmission [1, 7]. Instability of the CAG repeat length has also been reported in somatic tissues, with the largest expansion being observed in the brain [8, 9]. Cognitive decline, irritability and depression are often the first signs of disease, preceding clinical diagnosis and the development of motor symptoms [10]. Uncontrollable movements, difficulty in speech and swallowing lead to progressive physical deterioration, total dependency and need for full nursing care. Death is usually the result of secondary illness.

HTT is ubiquitously expressed. Neurodegeneration is the main HD phenotype, however, non-central nervous system HD-associated pathologies have also been reported [11–13]. Orthostatic hypotension, tachycardia, impaired modulation of cardiovascular tone and attenuated heart rate responses to stress, often present in early stage HD patients, have been ascribed to dysfunction of the autonomous nervous system [14–19]. Cardiac pathology, including atrophy, has been, however, described in HD murine models [13, 20–22]. In addition, cardiomyocyte-autonomous expression of 83 polyQ peptide in mouse model led to reduced cardiac function and dilatation by 5 months followed by death by 8 months. On the contrary, a 9-fold higher expression of 19 polyQ peptide in control animals had no effect on murine cardiac function or lifespan [23]. Taken together, the data from animal models suggest that the cardiac phenotype seen in HD patients is not exclusively a result of dysautonomia; and that the expression of mutant HTT in cardiomyocytes may also be cardiotoxic.

Human pluripotent stem cells, bearing the endogenous *HTT* mutation, can be differentiated into multiple cell lineages and kept in culture *ad infinitum*, circumventing the limitations associated with primary cell culture or immortalized cell lines with exogenously expressed mutant *HTT*. The aim of this study was to test the stability of CAG repeats in cardyomyocytes differentiated *in vitro* from three HD-hESC lines [24], KCL027, KCL028 and KCL036.

## Methods

### 2.1. hESC derivation, culture, expression of pluripotency markers and *in vivo* differentiation (teratoma)

HD-hESCs were derived from fresh embryos diagnosed with HD following Preimplantation Genetic Diagnosis (PGD). The work was done under the Human Fertilisation and Embryology Authority (HFEA; research license number R0133) and local ethical approval (UK National Health Service Research Ethics Committee Reference 06/Q0702/90) following the written informed consent from the donors. All hESC lines have been approved for deposit in the UK Stem Cell Bank by the Medical Research Council’s Steering Committee. All the lines are also listed in the NIH hESC Registry as ethically derived and eligible for use in NIH funded research. The methods describing hESC derivation, culture, expression of pluripotency markers and *in vivo* differentiation are reported in detail previously [25, 26].

### 2.2. Genotyping

Genotyping was performed as previously described [25]. Briefly, DNA was extracted from hESC cultures using a Chemagen DNA extraction robot, quantified using a Nanodrop spectrophotometer, and amplified using two multiplexes, one of 17 PCR primer pairs for markers on chromosomes 13, 18 and 21, and one of 14 primer pairs for markers on the X and Y chromosomes. PCR products, separated on an ABI3100 capillary genetic analyser, were analyzed using ABI Genotyper software. Allele sizes represent a unique fingerprint of each cell line.

### 2.3. Array comparative genomic hybridization (CGH)

Array CGH was performed as previously described [25]. Briefly, 1 μg DNA was labelled using a CGH labelling kit (Enzo Life Sciences, USA) and purified post-labelling using QIAquick PCR Purification Kit (Qiagen, USA). Labelling efficiency and yield was assessed by spectrophotometry (Nanodrop, USA). We used an Agilent (USA) 4 × 44 K platform with either Wessex NGRL design 017457 or design 028469. The data were processed using Feature Extraction and DNA Analytics software packages (Agilent, USA); 95% of array data was required to pass QC. For aberration calling, we used ADM-2 algorithm at threshold 6 (with a 3 probe sliding window providing a mean detection interval of 200 kb).

### 2.4. Cardiac differentiation

Directed cardiac differentiation was based on the work published by Laflamme *et al* [27]. Briefly, undifferentiated hESCs, maintained on Matrigel (BD) in Nutristem (Stemgent), were dissociated into a single-cell suspension by a 3–5 min incubation with accutase (STEMCELL Technologies). Cells were then centrifuged, resuspended, counted and seeded in Nutristem onto growth factor reduced Matrigel coated 24-well plates at a density of 2 x 10^5^ cells/cm^2^, with the addition of 10 μM Y-27632 (Source Bioscience) to support cell survival and attachment. This marked Day -5 of the differentiation protocol. Until Day 0, cells were fed daily with 2 ml/well of Nutristem. At Day 0, cardiac differentiation was induced by feeding the cells with 0.5 ml/well Roswell Park Memorial Institute medium (RPMI)-B27 medium (Invitrogen) supplemented with 100 ng/ml of human recombinant Activin A (Miltenyi). The following day, the medium was replaced by 1.5 ml/well RPMI-B27 supplemented with 10 ng/ml human recombinant BMP4 (Miltenyi) and left unchanged for four days. The medium was then exchanged for unsupplemented RPMI-B27 every two days for up to 60 days.

### 2.5. Reverse transcription (RT)-PCR and quantitative (q) PCR

Total RNA was extracted using the RNeasy Mini Kit (QIAGEN) following the manufacturer’s instructions. A 20 min DNAse I (27 Kunitz units final) (Qiagen) treatment step at room temperature was included to eliminate potential genomic DNA contamination. cDNA was generated by reverse-transcription of total RNA (500 ng) in a 20-μl reaction using the Precision nanoScript Reverse Transcription kit (PrimerDesign) following the manufacturer’s instruction. The qPCR reaction consisted of 5 μL of cDNA diluted 1:10 in water, 10 μl of Precision 2X qPCR Mastermix (PrimerDesign) and 300 μM final of each primer for a final volume of 20 μl. qPCR cycling conditions were as follow: 1 cycle 95°C for 10 min; 40 cycles 95°C for 15 s, 61°C for 30 sec followed by melt curve acquisition from 59°C to 95°C with 0.5°C increment. To select the best housekeeping gene, we tested a panel of 12 different housekeeping genes on undifferentiated hESCs and hESC-derived cardiomyocytes. GeNorm analysis was then performed via the qbase^PLUS^ software in order to determine the two most stable ones, which were *EIF4A2* and *SDHA*. The *POUF51* (also known as *OCT4*), *NANOG*, *TNNT2*, *EIF4A2* and *SDHA* primers were designed by PrimerDesign Ltd and the *HTT* primers were designed using the Primer3 website (*http*:*//frodo*.*wi*.*mit*.*edu/*). Primers are listed in S1 Table. All samples were analyzed in triplicate, Ct values were determined, and the expression was calculated by the 2^-ΔΔCt^ method. *EIF4A2* and *SDHA* were used for internal normalisation (S2 Table).

### 2.6. Isolation of DNA from formalin-fixed paraffin embedded (FFPE) teratoma tissue

Eight serial 6-μm sections from each paraffin block with embedded teratoma tissue were processed for the analysis. The first and the last section were stained with hematoxylin and eosin (H&E) and the tissue types spanning both sections were determined. We purified DNA from the remaining six sections and determined number of CAG repeats using human-specific primers. The wild-type (WT) allele was used as an internal control.

Isolation of DNA from FFPE tissue was adapted from Campos and Gilbert [29]. The FFPE tissue samples were digested in 500 μl alkali digestion buffer (0.1 M NaOH with 1% SDS, pH 12) for 40 min at 100°C. After a 5 min of cooling down period, 500 μl of 25:24:1 phenol:chloroform:isoamyl alcohol was added to the samples, followed by a 5 min agitation at room temperature and 5 min centrifugation at 10,000 x *g*. The upper aqueous layer was then removed and added to a new tube containing 500 μl choloroform. A second agitation and centrifugation step was performed before adding the aqueous layer to 1 volume of isopropanol and 0.1 volume of 3 M sodium acetate pH 5. The samples were then centrifuged 30 min at 10,000 x *g*. Liquid was then decanted and the DNA pellet washed twice with 1 ml 85% ethanol. After ethanol decantation, the DNA pellet was allowed to dry before resuspension in TE buffer.

### 2.7. CAG repeats

Preimplantation genetic haplotyping (PGH) for HD was performed as described previously [24, 25]. CAG repeat size determination of the cell (wells with predominantly beating cells) and tissue samples (teratoma sections) was performed by PCR as published [30]. Briefly, 5–50 ng DNA amplification was performed in 10 μl reactions containing: 0.2 mM dNTPs, 10% DMSO, AM buffer (67 mM Tris–HCl pH 8.8; 16.6 mM (NH_4_)SO_4_; 2 mM MgCl_2_; 0.17 mg/ml BSA), 0.8 pmol FAM-labelled forward primer (GAGTCCCTCAAGTCCTTCCAGCA) and reverse primer (GCCCAAACTCACGGTCGGT) and 0.5 U AmpliTaq DNA polymerase (Applied Biosystems). Cycling conditions were: 90 sec at 94°C, 35 × (30 sec at 94°C; 30 sec at 65°C; 90 sec at 72°C) and 10 min at 72°C. After the PCR, for each sample, 1 μl of PCR product was mixed with 9 μL of HiDi Formamide (Applied Biosystems) and 0.03 μl l MegaBACE ET900-R Size standards (GE Healthcare) and denatured at 95°C for 5 min. Samples were then run on an ABI3730 sequencer and analysed using GeneMapper v5.2- 3730XL software (Applied Biosystem).

Sensitivity of CAG assay was determined by mixing at different ratio KCL034 (clinical grade wild-type hESC line) and KCL027 (HD-hESC line) genomic DNA, each diluted exactly to 50 ng/ml (S1 Fig *)*.

## Results

### 3.1. Characterization of HD-hESC lines

The purpose of pre-implantation genetic diagnosis (PGD) is to identify and exclude embryos that carry the familial mutation. Such embryos are never destined for use in treatment and are routinely discarded as soon as an affected status is diagnosed. If the patients decide to donate these embryos to research, they give consent at the time of treatment. In our center we identify HD specific mutation-carrying embryos by haplotyping using polymorphic markers flanking and intragenic to the *HTT* gene on chromosome 4p16.3. Primers designed for 15 microsatellite markers enable testing for inheritance of the at-risk HD allele. Three HD-hESC lines, KCL027, KCL028 and KCL036, were derived in our center from embryos carrying HD-specific mutation from two donor couples. One couple had eight affected embryos from which two lines, KCL027 and KCL028, were derived and the other donor couple had one affected embryo, from which KCL036 was derived [24].

**Fig 1:**
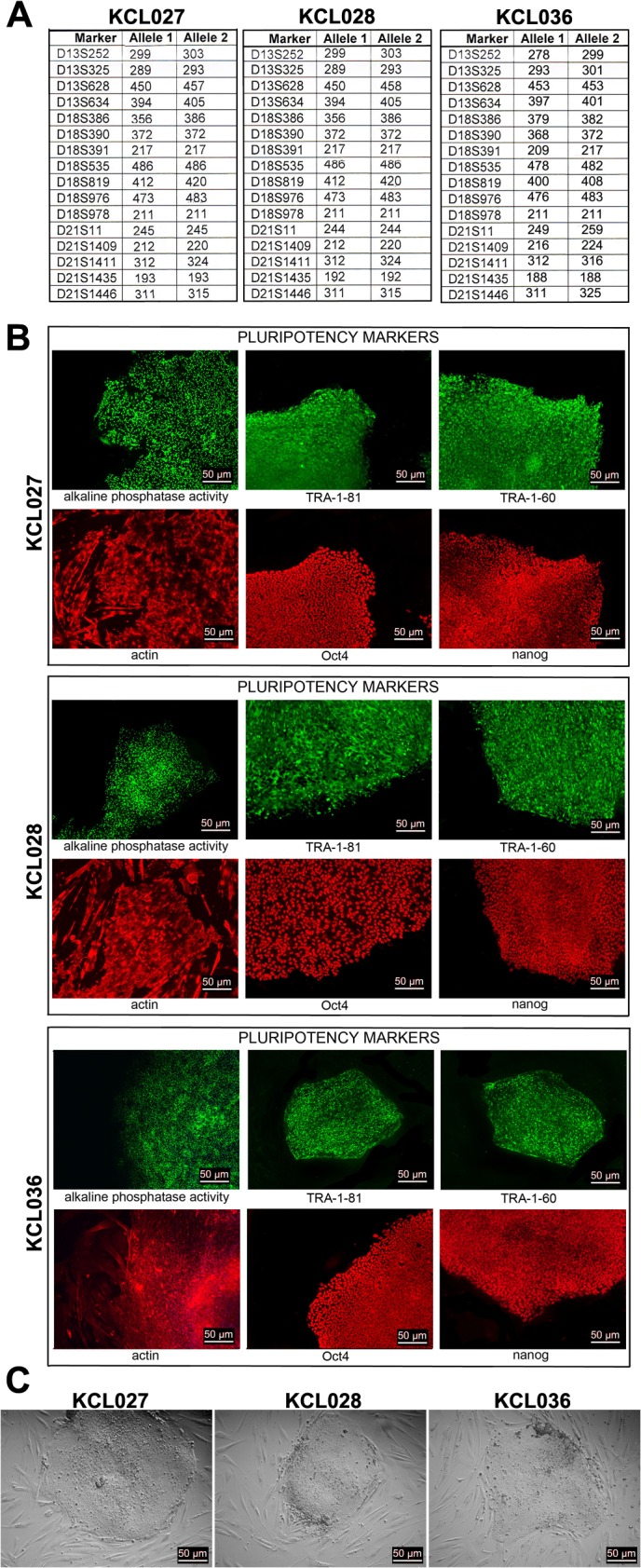
HD-hESC lines characterization. (A) Genotyping: Microsatellite markers specific for chromosomes 13, 18, 21, X and Y were amplified. The allele sizes in bp for markers on chromosomes 13, 18, and 21 are listed in the table. (B) Pluripotency markers: alkaline phosphatase (AP) activity, TRA-1–60, TRA-1–81, OCT4, and Nanog were detected in undifferentiated cells of all three hESC lines. (C) HD-hESC colonies have a typical morphology of normal hESC colonies.

All three lines were assessed for a number of parameters that are part of standard hESC lines characterization [26, 31]. The identity of the cell lines was confirmed by genotyping; amplification of polymorphic microsatellite markers was carried out, giving a unique fingerprint for each cell line (Fig 1A). The data confirmed that KCL027 and KCL028 are clonal related and are both unrelated to KCL036. The presence of pluripotency markers in all three lines was demonstrated by immunostaining using a panel of antibodies against transcription factors NANOG and OCT4 and cell surface antigens TRA-1-60 and TRA-1-81. The HD-hESC colonies were also positive for alkaline phosphatase (AP) activity using a commercially available kit (Fig 1B). They had a typical morphology of normal healthy hESC colonies (Fig 1C). As the first line test for genomic stability, we used array Comparative Genomic Hybridization (aCGH) as described previously [25]. aCGH is an oligonucleotide platform with 60,000 probes that can detect regions of imbalance down to approximately 25kb [32]. No genetic imbalance was detected during extended periods of culture (data not shown).

### 3.2. HD-hESC-derived cardiomyocytes have a stable number of CAG repeats

**Fig 2:**
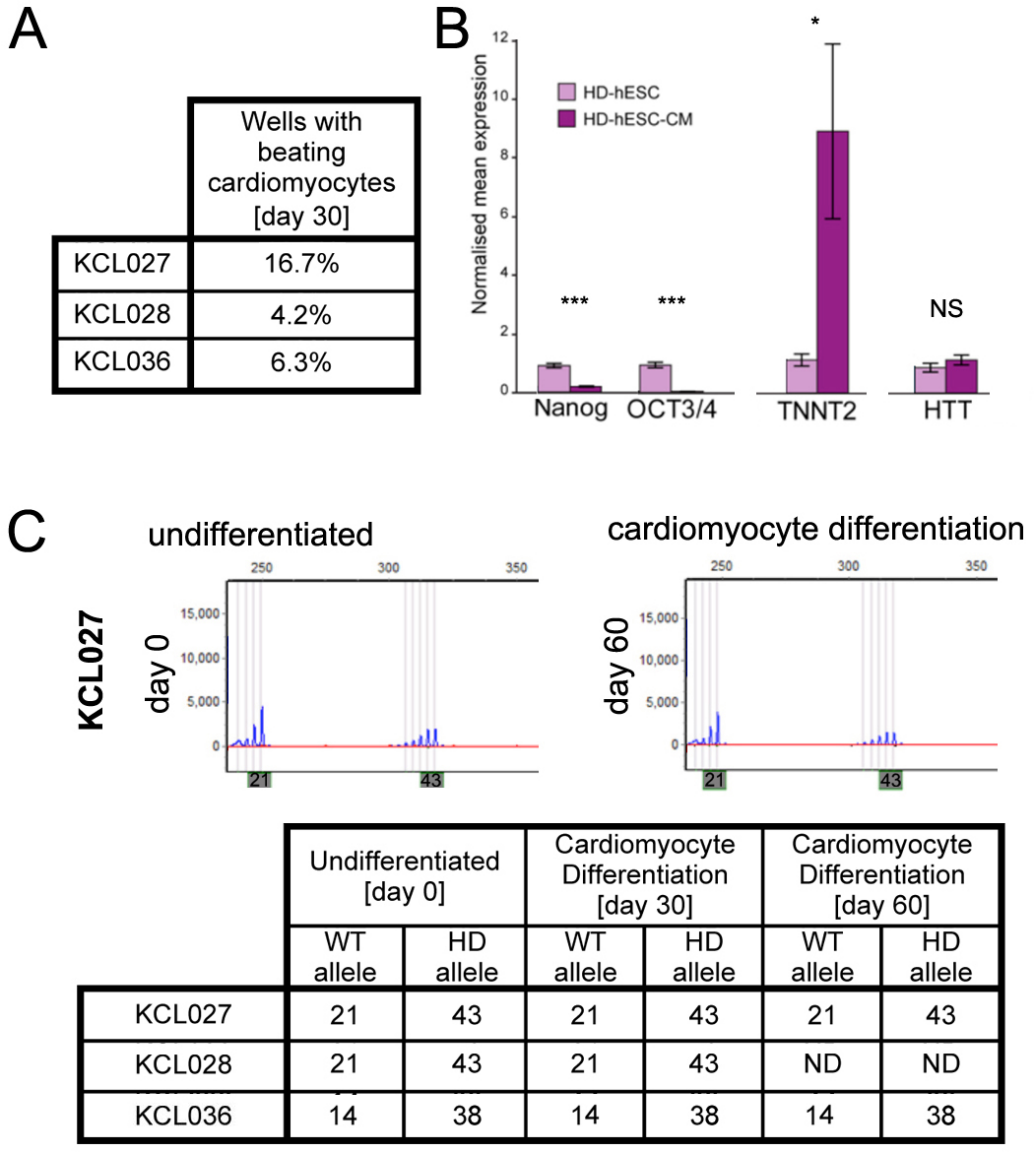
All three HD-hESC lines have stable number of CAG repeats upon directed cardiomyocyte differentiation in vitro. (A) Beating cardiomyocytes were present in all three HD hESC lines examined in 2 independent rounds of differentiation. (B) Upon differentiation into cardiomyocytes for 30 days, expression of the pluripotency markers *NANOG* and *OCT4* is nearly undetectable, whereas the cardiomyocyte marker *TNNT2* was increased. There is no change in *HTT* expression. Data are given by normalised mean ± standard error of the mean (n = 3); statistical significance was calculated by an unpaired homoscedastic one-tailed Student's t-test. NS, p ≥ 0.5 (non-significant); *p = 0.01–0.05 (significant); ***p ≤ 0.05 (extremely significant). (C) Top: Analysis of CAG repeats in undifferentiated KCL027 cells at the start of differentiation [day 0] and 60-day after. Bottom: Number of CAG repeats in allele carrying HD mutation has not increased after 30–60 days differentiation into cardiomyocytes.

Next, we asked whether directed differentiation into cardiomyocytes would result in a change in the number of CAG repeats. We differentiated the HD-hESCs in a monolayer following a directed differentiation protocol [27]. All three differentiated HD-hESC lines presented beating cardiomyocytes (Fig 2A). Using qPCR, we confirmed that 30 days after the start of differentiation, expression of pluripotency-associated genes *NANOG* and *OCT3/4* had diminished in all three hESC lines, whereas the expression of cardiac-associated gene *TNNT2* increased. *HTT* expression remained similar between undifferentiated hESCs and the cultures containing hESC-derived cardiomyocytes (Fig 2B). The CAG repeat number of all three HD-hESC lines in affected and non-affected alleles remained stable throughout differentiation (Fig 2C). We then extended culture of differentiated cardiomyocytes for an additional 30 days and showed that the number of CAG repeats in cells derived from two lines, KCL027 and KCL036, still remained unchanged (Fig 2C). To our knowledge, this is the first demonstration of differentiation of HD-mutation-carrying human pluripotent stem cells, either hESC or human induced pluripotent stem cells (hiPSC), into cardiomyocytes, and assessment of the effect on the number of the CAG repeats.

### 3.3. Number of CAG repeats remains stable in multiple *in vivo* differentiated tissues

**Fig 3:**
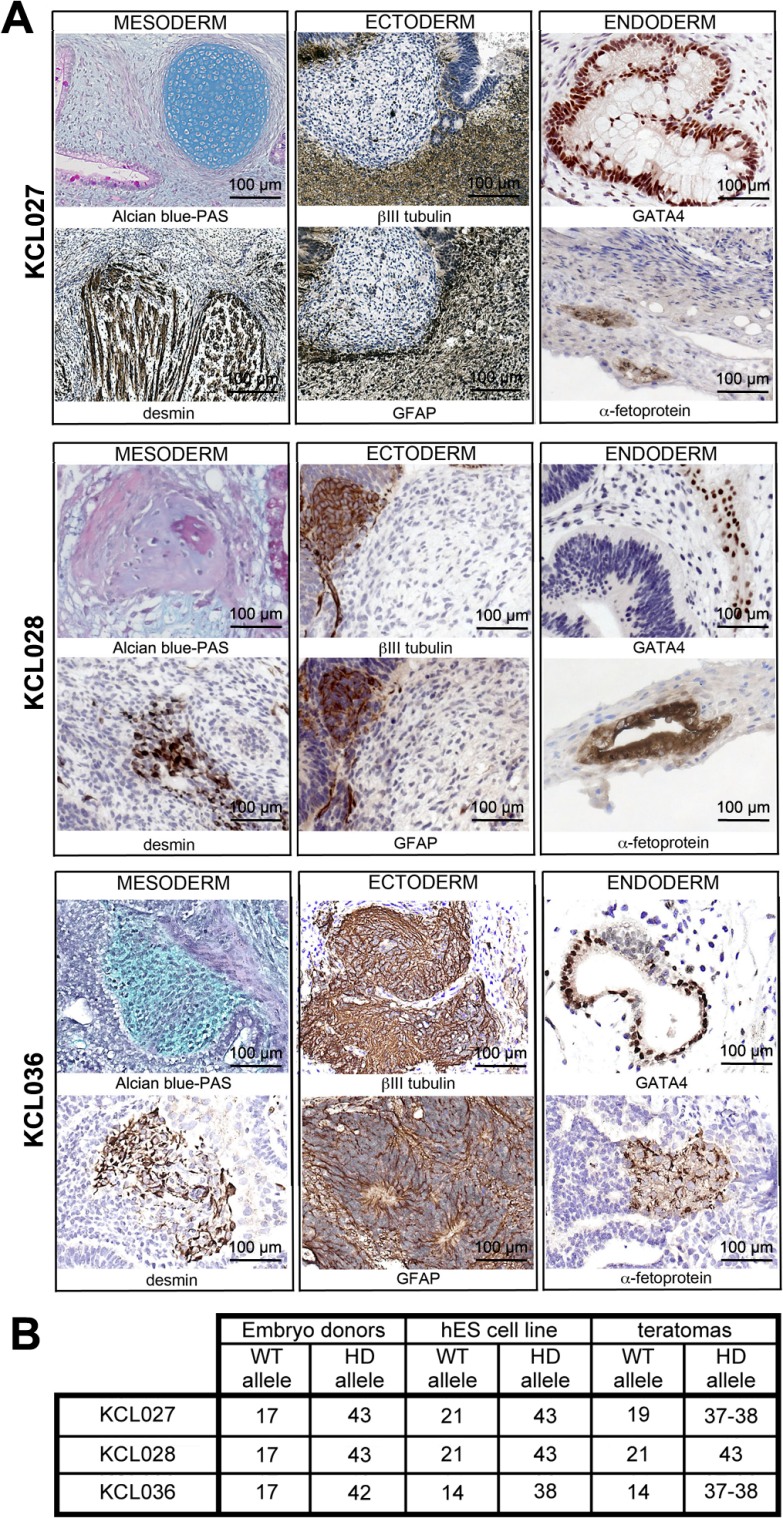
All three HD-hESC lines have stable number of CAG repeats upon spontaneous *in vivo* differentiation. (A) Differentiation into the three germ layers *in vivo*: Teratomas were encapsulated and did not invade surrounding tissue. Sections are counterstained with hematoxylin and eosin and specific stains are either light blue (Alcian blue) or brown (all immunohistochemistry). Germ layer markers: Alcian blue—and periodic acid–Schiff (PAS)-stained cartilage and desmin for mesoderm, neuron-specific β-III tubulin and glial fibrillary acidic protein (GFAP) for ectoderm, GATA4 and α- fetoprotein for endoderm. (B) Number of CAG repeats in allele carrying HD mutation did not increase during 10-week teratoma formation.

Since CAG stability might differ from one tissue to another, we allowed the hESC to differentiate spontaneously *in vivo* in order to mimic more closely a physiological environment. Different techniques of generating teratomas, encapsulated tumors with tissue derivatives of all three germ layers, have previously been described [33–35]. We injected cells embedded in Matrigel from each of three HD-hESC lines (KCL027, KCL028 and KCL036) subcutaneously into immunocompromized NOD-SCID mice (total n = 9 mice) and harvested them 10 weeks later. Animals homozygous for the SCID mutation have impaired T and B cell lymphocyte development, whereas the NOD background additionally results in deficient natural killer cell function. Teratomas in all lines consisted of tissue derivatives from all three germ layers (Fig 3A). All tumors contained glandular epithelium, immature neural epithelium, immature mesenchyme, cartilage and endothelium. In addition, KCL027 and KCL028 teratomas contained pigmented epithelium and arachnoidal tissue. We assessed the number of CAG repeats in DNA isolated from sections of FFPE teratoma tissue samples. We found no increase in the number of CAG repeats in the mutated allele in any of the lines examined (Fig 3B). However, we detected a slight decrease in the number of CAG repeats in both the WT and HD allele of line KCL027. Such short truncations have been reported previously [36, 37]. Taken together the data suggest that the CAG repeat length remains the same following two different differentiation techniques. To understand the molecular mechanisms of cardiac pathology in HD patients, HD-hESC and-hiPSC with a moderate increase in the number of CAG repeats may not be the best *in vitro* cellular model.

## Discussion

To complement HD *in vivo* models, HD *in vitro* models have been developed from mouse, rat and human cells. These are in general immortalized cell lines with a genome-integrating viral delivery system carrying a mutated *HTT* exon 1, expression of which is driven by an exogenous promoter. These cell lines recapitulated some of the hallmarks features of HD, notably an increased cell death and the formation of aggregates. Similar phenotypes have also been found in primary cell models. However, it is not until recently that stem cells have been used to model HD (Table 1).

**Table 1:** Pluripotent stem cell *in vitro* models of HD.

Cell line	Species origin	Cell type	CAG repeat size	Phenotype	Reference
	Mouse	Neural stem cells	140/140; *HTT* KO	Impaired motility, increased ROS, dysregulated cholesterol	[51]
HD-iPS1	Human	iPSC	72		[50, 52]
*Hdh*CAG	Mouse	ESC	7, 77 and 150	Large CAG repeat sizes increase neurogenesis	[53]
KCL005, KCL008	Human	ESC	46	Increase in CAG repeats upon differentiation into astroglial precursors	[41]
KCL012, KCL013, KCL027, KCL028, KCL036	Human	ESC	42, 46	No phenotype reported	[24, 25]
R6/1	Mouse	ESC	127	CAG expansion, impaired DNA repair, apoptosis	[54]
	Mouse	ESC	20, 50, 91, 111 and *HTT* KO	Dysregulation of 73 genes	[55]
TrES1	Monkey	Pluripotent SC generated by the fusion of transgenic HD monkey skin fibroblast and a wild-type monkey oocyte	84	Presence of aggregates	[56]
rHD-ESC	Monkey	ESC			[57]
	Human	ESC with exogenous exon1 expression?		Insoluble HTT aggregates and neurodegeneration	[58]
SI-186, SI-187	Human	ESC	37 and 51	Increase in CAG repeats upon neural differentiation; elevated glutamate-evoked responses	[39, 40]
SI-187, SIVF017, SIVF018, SIVF020, Huez2.3, VUB05	Human	ESC	40–51	Downregulation of HTT, dysregulation of CHCHD2, TRIM4, and PKIB	[42–44]
HD-iPS^hom^ 4F-1, HD-iPS^hom^ 4F-2, HD-iPS^hom^ 4F-3, HD-iPS^hom^ 3F-1, HD-iPS^hom^ 3F-2, HD-iPS^het^ 3F-1	Human	iPSC	42/44, 39/42, 45	Increase in lysosomal activity	[47]
HD60n, HD60i.3, HD60i.4, HD109i.1, HD180n, HD180i.5, HD180i6, HD180i7	Human	iPSC	60, 109, 180	Disease-associated changes in electrophysiology, metabolism, cell adhesion, and apoptosis	[46]
	Human	iPSC	72	Deregulated expression of oxidative stress proteins; aggregates	[48, 49]

Since the first disease-specific hESC line was derived from patients undergoing PGD for cystic fibrosis [38], more than 20 HD-hESC lines have been derived worldwide. However, only a few of them have been thoroughly characterized or used as research tools. Two HD-hESC lines carrying 37 and 51 CAG repeats were indistinguishable morphologically from normal control cell lines in the undifferentiated state and during differentiation into forebrain neurons. However, neural differentiation induced instability of CAG repeats and lines were gaining five to six CAG repeats upon differentiation [39]. The expression of those genes that are deregulated in HD remained unperturbed throughout differentiation. The only phenotype observed was an elevated glutamate-evoked response in neurons differentiated from an HD-hESC line with 51 CAG repeats [40]. A marked increase in the number of CAG repeats from 46 to 70 upon differentiation into astroglial precursors was found in one HD-hESC line derived in our laboratory [41]. On the other hand, two other groups reported that 40–48 CAG repeats were stable in five undifferentiated and differentiated HD-hESCs [42, 43]. A later study involving these five HD-hESC lines reported a down-regulation of the *HTT* gene itself in HD neural cells and dysregulation of three genes, of which two, *CHCHD2* and *TRIM4*, were also dysregulated in blood cells from the HD patient [44]. In contrast, we could not detect any change in the levels of *HTT* gene expression in the cell exposed to our cardiac differentiation protocol (Fig 2B), indicating that this phenomenon might be either cell line-related or specific to HD neurons but not to other cell types. We also found that the number of CAG repeats was stable in cells that underwent undirected in vivo differentiation, and those that underwent directed in vitro differentiation towards a cardiac lineage. Taking together the reports from different groups to date, it seems that some lines are prone to CAG expansion *in vitro* and others are not. Molecular mechanisms behind these differences are still unknown. Although cell culture conditions might play a role, genetic background of each individual line is likely to be more important. It would be interesting to see how unstable CAG repeats in cell lines correlate with anticipation in the families that donated the embryos. However, confidentiality surrounding human embryo donation makes such comparison problematic.

Reprogramming of somatic cells into iPSC, discovered by Takahashi and Yamanaka [45], could circumvent these issues. Derivation of HD-iPSC lines will enable comparison of disease-in-the-dish phenotypes with clinical findings in somatic cell donors and affected members of their families, leading to a better understanding of the role of genetic background and molecular mechanisms in development of HD-associated symptoms. The HD iPSC consortium recently published findings showing that the positive correlation between the sizes of CAG repeats and the severity of symptoms observed in HD patients could also be observed *in vitro* in HD-iPSC derived neurons [46]. A significant increase in lysosomal activity both during self-renewal and in iPSC-derived neurons has been noted in the iPSC lines derived from two homozygotes carrying 42/44 and 39/42 CAG repeats and one heterozygote carrying 17/45 CAG repeats [47]. The number of CAG repeats remained stable during the extended period of culture. Another HD-iPSC line with 72 CAG repeats did not show any phenotype when cultured *in vitro* or differentiated into neural precursors. However, when either subjected to oxidative stress [48], proteasome inhibitor or engrafted into neonatal brains for 33 weeks, the HD-iPSCs exhibited signs of HD pathology [49]. Elevated caspase activity in HD-iPSCs was also detected in derived neurons of HD-iSPC line with 72 repeats [50].

The CAG-repeat expansion associated phenotypes already reported in transgenic models of HD are the result of exogenous mutation, whereas phenotypes in HD-hESC and HD-iPSC would be caused by endogenous mutation, more closely modeling *in vivo* disease. Our data further highlight the need to generate HD-iPSCs with a wider range of repeats and to correlate the repeat instability in different tissue types with the medical history of donors and affected members of their families.

## Supporting Information

S1 Fig The assay that we were using to detect abnormal number of CAG repeats in one *HTT* allele could detect the mutation present in 1 μl of DNA from HD-hESC line KCL027 at concentration 50 ng/ml mixed with 39 μl of normal wild-type hESC line KCL034 of the identical concentration, which would equal 1 out of 40 cells (2.5%). (EPS)

S1 Table Sense and Antisense primers of target genes used for hESC-derived cardiomyocytes characterization. (DOCX)

S2 Table Housekeeping genes designed and synthesized by PrimerDesign Ltd used for hESC-derived cardiomyocyte characterisation amplicon context. The primer sequences are commercially sensitive information but the details provided in this table are MIQE compliant [28].
(DOCX)
