# Supplementary material for: Three Huntington’s Disease Specific Mutation-Carrying Human Embryonic Stem Cell Lines Have Stable Number of CAG Repeats upon In Vitro Differentiation into Cardiomyocytes
Source: PLoS One. 2015 May 20;10(5):e0126860. doi: 10.1371/journal.pone.0126860 (PMC4438866; doi:10.1371/journal.pone.0126860)
Supplement: S1 Table — (DOCX) [file pone.0126860.s002.docx]

| **Gene** | **Accession number** | **Sense Primer** | **Anti-sense primer** |
| --- | --- | --- | --- |
| *OCT4* | NM_002701 | GCCGTGAAGCTGGAGAAG | GTGTATATCCCAGGGTGATCC |
| *NANOG* | NM_024865 | ATGTCTTCTGCTGAGATGCC | GTTGTTTGCCTTTGGGACTG |
| *TNNT2* | NM_000364 | CAAAGCCCAGGTCGTTCAT | GCAACTCATTCAGGTCCCTTCT |
| *HTT* | NM_002111 | TCTGGGCATCGCTATGGAAC | ATTTCTGAGGCCGAACCAGG |

**Supplemental Table S1:** Sense and antisense primers of target genes used for HD-hESC-derived cardiomyocytes characterization**.**
