# Supplementary material for: Three Huntington’s Disease Specific Mutation-Carrying Human Embryonic Stem Cell Lines Have Stable Number of CAG Repeats upon In Vitro Differentiation into Cardiomyocytes
Source: PLoS One. 2015 May 20;10(5):e0126860. doi: 10.1371/journal.pone.0126860 (PMC4438866; doi:10.1371/journal.pone.0126860)
Supplement: S2 Table — The primer sequences are commercially sensitive information but the details provided in this table are MIQE compliant [28]. (DOCX) [file pone.0126860.s003.docx]

| **Gene** | **Accession number** | **Anchor nucleotide:** | **Amplicon length** |
| --- | --- | --- | --- |
| *EIF4A2* | NM_001967 | 900 | 113 |
| *SDHA* | NM_004168 | 1032 | 120 |

**Supplemental Table S2:** Housekeeping genes designed and synthesized by PrimerDesign Ltd used for HD-hESC-derived cardiomyocyte characterisation amplicon context.

The primer sequences are commercially sensitive information but the details provided in this table are MIQE compliant.^28^
